# Epidemics, pandemics and income inequality

**DOI:** 10.1186/s13561-022-00355-1

**Published:** 2022-01-18

**Authors:** Chrys Esseau-Thomas, Omar Galarraga, Sherif Khalifa

**Affiliations:** 1 International Monetary Fund, Washington, DC USA; 2 Brown University, Providence, RI 02912 United States; 3 California State University, Fullerton, CA 92834 USA

**Keywords:** Epidemics, Executive, Income inequality, I14, D31

## Abstract

The novel coronavirus is part of a series of infectious disease outbreaks that include: Ebola, Avian influenza, Middle East respiratory syndrome coronavirus, and Influenza A. This paper addresses the question of how do these epidemics and pandemics affect income inequality in countries around the world during the first two decades of this century. To achieve its objective, the paper develops a model that indicates a positive association between these health crises and income inequality. To empirically test our theoretical predictions, the paper explores the effect on the Gini coefficient of a dummy variable that indicates the occurrence of an epidemic or a pandemic in a country in a given year and the number of deaths per 100,000. To properly address potential endogeneity, we implement a Three-Stage-Least Squares technique. The estimation shows that the number of deaths per 100,000 population variable has a statistically significant positive effect on the Gini coefficient, especially when we incorporate COVID-19 data. This suggests that not only the occurrence, but also the health consequences of COVID-19 have a significant and economically important effect on income inequality.

**Background:** The purpose of the study is to examine the effect of epidemics and pandemics on income inequality. This has important implications as the outcome of this study can guide policymakers into implementing policies that can mitigate the economic consequences of these health crises.

**Methods:** The study is a cross country analysis using fixed effects estimation. To address potential endogeneity and determine causality, the paper uses the Three-Stage-Least-Squares estimation.

**Results:** The paper finds that the number of epidemic deaths per 100,000 population variable has a statistically significant positive effect on the Gini coefficient, especially when we incorporate COVID-19 data.

**Conclusions:** The paper finds that it is not only the occurrence of an epidemic, captured by the epidemics dummy variable, but also the health consequences, captured by the number of deaths per 100,000 population, that have a significant effect on income inequality. This is especially the case when we incorporate COVID-19 in our analysis.

**Trial registration: **Not Applicable.

## Introduction

This paper examines the effect of epidemics and pandemics on income inequality. An epidemic is a widespread outbreak of an infectious disease in a community. A pandemic is an epidemic that has spread worldwide. This paper argues that epidemics and pandemics have significant effects on income distribution either through their potential direct disproportionate health effects, or through their indirect economic consequences caused by the intervention of governments to contain these health crises. This question is important as policy makers around the world attempt nowadays to comprehend and address the consequences of emerging disease epidemics and pandemics.

To achieve its objective, the paper develops a simple theoretical setup extending the framework in Moser and Yared [18] to one with low skilled workers and high skilled workers. In this context, policy makers can impose a lockdown to contain an epidemic or a pandemic. The lockdown policy imposes a cap on the supply of low skilled workers and causes more high skilled workers to work remotely. The model shows that there is a threshold lockdown policy beyond which income inequality increases. To empirically test the theoretical findings, the paper uses a panel of 191 countries during the period 2000–2020 to examine the effect of epidemics and pandemics on income inequality. During this period, the world experienced several outbreaks of infectious diseases such as MERS-Cov, H1N1, SARS, Ebola and SARS-Cov-2. In this context, we use a dummy variable to indicate the occurrence of an epidemic or a pandemic in a country in a given year, in addition to the number of deaths per 100,000 population. Panel estimation techniques are used to explore the association between these health indicators and the Gini Coefficient.

In this context, the paper addresses the issue of potential endogeneity. As much as epidemics and pandemics can affect income distribution, the extent of income inequality can determine the disproportionate exposure of various groups to a health crisis causing it to exacerbate to an epidemic or a pandemic. To properly address the issue of causality, we use a Three-Stage-Least Squares estimation technique. The estimation considers several control variables that are identified by the literature as determinants of income inequality, such as the level of economic development captured by the logarithm of GDP per capita, the sectoral structure of the economy, the institutional quality captured by the extent of corruption and the democratic system of governance, in addition to indicators of fiscal policy, monetary policy and trade policy.

The fixed effects estimation shows that the dummy variable has a robust and statistically significant positive effect on income inequality, while the number of deaths per 100,000 population has an insignificant effect. The interpretation is that it is the occurrence of a pandemic, captured by the dummy variable, that is what triggers the policy response that may affect income inequality, rather than the health consequences of these crises, captured by the number of deaths per 100,000. To properly address potential endogeneity, we implement a Three-Stage-Least Squares estimation technique. The 3SLS estimations show that the number of deaths per 100,000 population variable has a statistically significant positive effect on the Gini coefficient, especially when we incorporate the effect of COVID-19. This suggests that not only the occurrence but also the health consequences of COVID-19 have a significant effect on income inequality.

The remainder of the paper is organized as follows: section 2 contains the literature survey, section 3 contains the theoretical model, section 4 discusses the intuition, section 5 on methods includes (a) the description of the data, and (b) the empirical estimation, section 6 presents the baseline results and robustness tests, second 7 includes a final discussion, and section 8 concludes. References, tables and figures are included thereafter.

## Literature

This paper contributes to a nascent literature on the economic consequences of epidemics and pandemics. Studies in this literature focus primarily on the effects of these health crises on labor market outcomes, income distribution and aggregate economic conditions. For instance, Lin and Meissner [16] find that stay-at-home orders are only weakly associated with slower growth of COVID-19 cases, while job losses have been no higher in U.S. states that implemented stay-at-home orders during the COVID-19 pandemic than others that did not. Rojas et al. [22] estimate how the spread of COVID-19 and school closures affect labor market conditions at the state level. The authors find that the slowdown in the labor market was due to the nationwide reaction, while state policies and epidemiological conditions have had a modest effect. Papanikolaou and Schmidt [20] analyze the supply-side disruptions of COVID-19. The authors find that sectors in which a larger fraction of the workforce is not able to work remotely experienced greater declines in employment, greater declines in expected revenue growth, worse stock market performance, and higher expected likelihood of default.

Borjas and Cassidy [5] show that the employment decline due to COVID-19 was severe for immigrants who had previous employment advantage in the labor market. This is because immigrants were less likely to work in jobs that could be performed remotely during the lockdown. Montenovo et al. [17] show greater declines in employment during the COVID-19 pandemic for Hispanics, young workers, those with high school degrees and some college, and those with occupations that require more interpersonal contact. Forsythe et al. [11] find that the collapse in job vacancies and the spike in unemployment insurance claims due to Covid-19 affected all the states of the United States, irrespective of the intensity of the spread of the virus or the timing of stay-at-home policies. The authors also show that nearly all industries and occupations saw contraction in job postings and increases in unemployment insurance claims, with the exception of essential front line jobs.

Sumner et al. [24] show that COVID-19 poses a challenge to the United Nations Sustainable Development Goal of ending poverty by 2030 as “global poverty could increase for the first time since 1990 demonstrating a reversal of a decade of progress in reducing world poverty.” The authors show that under the scenario of a 20% income or consumption contraction, the number of people living in poverty could increase by 420–580 million, compared to 2018. Fenichel [10] compare social distancing outcomes under decentralized, full control social planner, and constrained social planner. The author finds that constrained social planner decision making can in some instances make society worse off than decentralized decision making with no intervention.

Some studies also discuss the distributional consequences of epidemics. For instance, Bell and Gersbach [3] find that during an outbreak, programs under which supported families enjoy the benefits of spending on health and education simultaneously are superior to those under which the benefits are sequenced. The authors also find that these superior programs restrict support to fewer families initially and thus increases income inequality. Glover et al. [14] develop a model that predicts that young workers in sectors deemed non-essential would benefit from ending the shutdown, while others would lose. The authors conclude that redistribution can render the welfare impacts less unequal. Furceri et al. [12] show that the COVID-19 pandemic could exert an adverse effect on income inequality in the absence of efforts to protect the vulnerable segments of society. The authors conclude that their finding that inequality increases with the adverse effect of a pandemic on economic activity “suggests that, all else equal, the distributional consequences of COVID-19 may be larger than those in previous pandemic episodes.” Palomino et al. [19] explore the effect of lockdowns on income inequality and poverty in Europe. The authors find that a two months’ lockdown scenario causes an average increase in the headcount poverty index of 4.9 percentage points, and an increase in the Gini coefficient by 3.5 percentage point. The authors also show that these estimates increase under longer lockdown scenarios and that both between-countries and within-countries income inequality significantly increase, with the change of the latter being more important.

Our paper’s contribution to the literature is threefold. It is the first to have a global analysis of the effects of epidemics and pandemics on income inequality, it is the first to address the issue of causality between these health crises and income inequality, and it is also the first to develop a simple theoretical framework that helps us understand the effect of these health crises on income inequality.

## Model

Consider an infinite-horizon model of an economy during a pandemic. Periods are indexed by *t* = 0, 1, . …. The economy is populated by a continuum of low skilled agents of unit mass, and a continuum of high skilled agents of unit mass. In each period, policy makers choose a lockdown policy {*L*_*t*_, *H*_*t*_}. *L*_*t*_ determines the fraction of low skilled labor who cannot work, while *H*_*t*_ determines the fraction of high skilled labor who can work remotely. This lockdown policy affects economic output negatively but curbs the spread of disease. Second, labor is supplied such that the wage equals the marginal product of labor. Third, production takes place and proceeds are paid to firms and workers. Finally, the pandemic evolves according to a model of disease spread, which depends on the lockdown policy. Thus, this model is an extension of that of Moser and Yared [18] to a setup where we introduce two types of labor and with a primary focus on the effect of pandemic lockdown policy on income inequality.

The production function states that output depends on the supply of low skilled labor *l*_*t*_, high skilled labor *h*_*t*_, and Ω_*t*_ which is the health state at date *t*. Thus, the production function is as follows

1 $$ {y}_t=f\left({l}_t,{h}_t,{\Omega}_t\right) $$

We assume that the function *f*(.) is continuously differentiable, increasing, and globally concave in *l*_*t*_ and *h*_*t*_. Thus, $li{m}_{l\to 0}\left(\frac{\partial f}{\partial l}\right)= li{m}_{h\to 0}\left(\frac{\partial f}{\partial h}\right)=\infty$ and $li{m}_{l\to \infty}\left(\frac{\partial f}{\partial l}\right)= li{m}_{h\to \infty}\left(\frac{\partial f}{\partial h}\right)=0$. The dependence of the production function on the health state reflects the possibility that the pandemic could decrease output by debilitating the labor force, by changing the share of the labor force working from office and from home, and by adopting social distancing efforts that may affect production.

The policy makers choose a lockdown policy *L*_*t*_ ∈ [0, 1] which reflects the fraction of low skilled labor that is prohibited from working. If *L*_*t*_ =0, there is no lockdown and all low skilled agents can go to work. If *L*_*t*_ =1, there is maximal lockdown and no agent is allowed to work. Low skilled agents inelastically supply effective labor *l*_*t*_ up to an upper bound $\left(1-{L}_t\right)\overline{l}\left({\Omega}_t\right)$. The lockdown policy also determines *H*_*t*_ which is the fraction of high skilled labor that will work remotely. If *H*_*t*_ =0, there is no lockdown and all high skilled agents can go to work in-person. If *H*_*t*_ =1, there is maximal lockdown and all high skilled agents are only allowed to work remotely. High skilled agents inelastically supply effective labor *h*_*t*_ up to an upper bound $\left(1-{H}_t\right)\overline{h}\left({\Omega}_t\right)+{H}_t\overset{\sim }{h}\left({\Omega}_t\right)$. In this context, those working in-person supply $\overline{h}\left({\Omega}_t\right)$ while those working remotely supply $\overset{\sim }{h}\left({\Omega}_t\right)$, where $\overset{\sim }{h}\left({\Omega}_t\right)>$ $\overline{h}\left({\Omega}_t\right)$ ∀*t*. This is because those who work remotely will be able to supply more labor as they save on the time they spend commuting to work in the absence of a lockdown. Thus, the labor market clearing conditions are as follows

2 $$ {l}_t=\left(1-{L}_t\right)\overline{l}\left({\Omega}_t\right) $$

3 $$ {h}_t=\left(1-{H}_t\right)\overline{h}\left({\Omega}_t\right)+{H}_t\overset{\sim }{h}\left({\Omega}_t\right) $$

Firms maximize profits *π*_*t*_ such that

4 $$ {\pi}_t={y}_t-{w}_t^l{l}_t-{w}_t^h{h}_t $$

where the price of output is normalized to 1, ${w}_t^l$ is the wage of low skilled labor, and ${w}_t^h$ is the wage of high skilled labor. Labor is competitively supplied so wages equal the marginal product of labor as follows

5 $$ {w}_t^l=\frac{\partial f\left({l}_t,{h}_t,{\Omega}_t\right)}{\partial {l}_t} $$

6 $$ {w}_t^h=\frac{\partial f\left({l}_t,{h}_t,{\Omega}_t\right)}{\partial {h}_t} $$

In this context, workers consume their wage income

7 $$ {c}_t^l={w}_t^l{l}_t $$

8 $$ {c}_t^h={w}_t^h{h}_t $$

where ${c}_t^l$ is the aggregate consumption of low skilled labor, and ${c}_t^h$ is the aggregate consumption of high skilled labor. In this context, social welfare equals the discounted sum of utility streams as follows

9 $$ {\sum}_{t=0}^{\infty }{\beta}^tu\left({c}_t^l,{c}_t^h,{\Omega}_t\right) $$

where *β* ∈ (0, 1) is the discount factor, and *u*(. ) is a strictly increasing and strictly concave utility function of consumption ${c}_t^l$ and ${c}_t^h$ and the health state Ω_*t*_. In this economy, income inequality can be captured by the share of income of high skilled labor to the total income of low skilled and high skilled labor as follows

10 $$ \frac{w_t^h{h}_t}{\left({w}_t^l{l}_t+{w}_t^h{h}_t\right)} $$

Finally, we model disease spread following a Susceptible-Infected-Recovered-Dead (SIRD) model as in Moser and Yared [18]. This model defines a law of motion of the health state as follows

11 $$ {\Omega}_{t+1}=\Gamma \left({H}_t,{L}_t,{\Omega}_t\right) $$

which depends on the degree of lockdown at date *t*. The initial health state Ω_0_ is taken as given. This setup allows us to derive some conclusions on the relationship between the pandemic lockdown policy and income inequality.

### Proposition 1

*There is a threshold H*^∗^ *such that* $\left(\frac{\partial {w}_t^h{h}_t}{\partial {h}_t}\right)>0$*,* ∀*H* > *H*^∗^ *if and only if* $\left(\frac{\partial {w}_t^h}{\partial {h}_t}\right)<1$ *at H* = 1*.*

**Proof.** At *H* = 0, we have to consider three possibilities: either $\left(\frac{\partial {w}_t^h}{\partial {h}_t}\right)>1$, $\left(\frac{\partial {w}_t^h}{\partial {h}_t}\right)=1$ or $\left(\frac{\partial {w}_t^h}{\partial {h}_t}\right)<1$. (1) If $\left(\frac{\partial {w}_t^h}{\partial {h}_t}\right)>1$ at *H* = 0 and $\left(\frac{\partial {w}_t^h}{\partial {h}_t}\right)<1$ at *H* = 1. Given that the marginal product of high skilled labor decreases with an increase in the supply of high skilled labor since $li{m}_{h\to \infty}\left(\frac{\partial f}{\partial h}\right)=0$, then there exists a threshold *H*^∗^ ∈ (0, 1) where $\left(\frac{\partial {w}_t^h}{\partial {h}_t}\right)=1$. This implies that $\left(\frac{\partial {w}_t^h{h}_t}{\partial {h}_t}\right)>0$, ∀*H* > *H*^∗^. (2) If $\left(\frac{\partial {w}_t^h}{\partial {h}_t}\right)=1$ at *H* = 0 then *H*^∗^ = 0. (3) If $\left(\frac{\partial {w}_t^h}{\partial {h}_t}\right)<1$ at *H* = 0 then $\left(\frac{\partial {w}_t^h{h}_t}{\partial {h}_t}\right)>0$ ∀ *H*.

### Proposition 2

*There is a threshold L*^∗^ *such that* $\left(\frac{\partial {w}_t^l{l}_t}{\partial {l}_t}\right)<0$*,* ∀*L* > *L*^∗^ *if and only if* $\left(\frac{\partial {w}_t^l}{\partial {l}_t}\right)<1$ *at L* = 1*.*

**Proof.** At *L* = 0, we have to consider three possibilities: either $\left(\frac{\partial {w}_t^l}{\partial {l}_t}\right)>1$, $\left(\frac{\partial {w}_t^l}{\partial {l}_t}\right)=1$ or $\left(\frac{\partial {w}_t^l}{\partial {l}_t}\right)<1$. (1) If $\left(\frac{\partial {w}_t^l}{\partial {l}_t}\right)>1$ at *L* = 0 and $\left(\frac{\partial {w}_t^l}{\partial {l}_t}\right)<1$ at *L* = 1. Given that the marginal product of low skilled labor increases with a decrease in the supply of low skilled labor since $li{m}_{l\to 0}\left(\frac{\partial f}{\partial l}\right)=\infty$, then there exists a threshold *L*^∗^ ∈ (0, 1) where $\left(\frac{\partial {w}_t^l}{\partial {l}_t}\right)=1$. This implies that $\left(\frac{w_t^l{l}_t}{\partial {l}_t}\right)<0$, ∀*L* > *L*^∗^. (2) If $\left(\frac{\partial {w}_t^l}{\partial {l}_t}\right)=1$ at *L* = 0 then *L*^∗^ = 0. (3) If $\left(\frac{\partial {w}_t^l}{\partial {l}_t}\right)<1$ at *L* = 0 then $\left(\frac{w_t^l{l}_t}{\partial {l}_t}\right)$ <0 ∀ *L*.

### Proposition 3

*The pandemic lockdown policy increases income inequality if H* > *H*^∗^ *and L* > *L*^∗^*.*

**Proof.** If *H* > *H*^∗^, then $\left(\frac{\partial {w}_t^h{h}_t}{\partial {h}_t}\right)>0$ and if *L* > *L*^∗^, then $\left(\frac{\partial {w}_t^l{l}_t}{\partial {l}_t}\right)<0$. This implies that the lock down policy increases *h*_*t*_ and decreases *l*_*t*_, then ${w}_t^h{h}_t$ increases if *H* > *H*^∗^ while ${w}_t^l{l}_t$ decreases if *L* > *L*^∗^. This causes an increase in income inequality captured by $\left(\frac{w_t^h{h}_t}{w_t^l{l}_t+{w}_t^h{h}_t}\right)$.

**Proposition 4** *H*^∗^ *decreases with an increase in the slope* $\frac{\partial {h}_t}{\partial {H}_t}=\left(\overset{\sim }{h}-\overline{h}\right)$*.*

**Proof.** At *H*^∗^, $\left(\frac{\partial {w}_t^h{h}_t}{\partial {h}_t}\right)=0$. We have

$$ {w}_t^h{h}_t=\left[\frac{\partial f(.)}{\partial {h}_t}\right]{h}_t=\frac{\partial f(.)}{\partial {h}_t}\left[\left(1-{H}_t\right)\overline{h}+{H}_t\overset{\sim }{h}\right] $$

In this case,

$$ \left(\frac{\partial {w}_t^h{h}_t}{\partial {h}_t}\right)=\frac{\partial^2f(.)}{\partial {h}_t}\left[\overline{h}+\left(\overset{\sim }{h}-\overline{h}\right){H}_t\right]+\frac{\partial f(.)}{\partial {h}_t} $$

which is equal to 0 at *H*^∗^. Thus,

$$ \left(\frac{\partial {w}_t^h{h}_t}{\partial {h}_t}\right)=\frac{\partial^2f(.)}{\partial {h}_t}\left[\overline{h}+\left(\overset{\sim }{h}-\overline{h}\right){H}_t^{\ast}\right]+\frac{\partial f(.)}{\partial {h}_t}=0 $$

This implies that in order to maintain the equality, ${H}_t^{\ast }$ is lower with a higher slope $\left(\overset{\sim }{h}-\overline{h}\right)$. A lower ${H}_t^{\ast }$ implies that income inequality can increase with a smaller fraction of high skilled labor working remotely.

**Proposition 5** *L*^∗^ *decreases with an increase in the slope* $\frac{\partial {l}_t}{\partial {L}_t}=-\overline{l}$*.*

**Proof.** At *L*^∗^, $\left(\frac{\partial {w}_t^l{l}_t}{\partial {l}_t}\right)=0$. We have

$$ {w}_t^l{l}_t=\left[\frac{\partial f(.)}{\partial {l}_t}\right]{l}_t=\frac{\partial f(.)}{\partial {l}_t}\left[\left(1-{L}_t\right)\overline{l}\right] $$

In this case,

$$ \left(\frac{\partial {w}_t^l{l}_t}{\partial {l}_t}\right)=\frac{\partial^2f(.)}{\partial {l}_t}\left[\left(1-{L}_t\right)\overline{l}\right]+\frac{\partial f(.)}{\partial {l}_t} $$

which is equal to 0 at *L*^∗^. Thus,

$$ \left(\frac{\partial {w}_t^l{l}_t}{\partial {l}_t}\right)=\frac{\partial^2f(.)}{\partial {l}_t}\left[\left(1-{L}_t^{\ast}\right)\overline{l}\right]+\frac{\partial f(.)}{\partial {l}_t}=0 $$

This implies that in order to maintain the equality, ${L}_t^{\ast }$ is lower with a higher slope $-\overline{l}$. A lower ${L}_t^{\ast }$ implies that income inequality can increase with a smaller fraction of low skilled labor not working.

### Proposition 6

*A higher level of income inequality decreases social welfare if and only if H* > *H*^∗^ *and L* > *L*^∗^ *and if* $\left|\frac{\partial {w}_t^l{l}_t}{\partial {l}_t}\right|>\left|\frac{\partial {w}_t^h{h}_t}{\partial {h}_{t.}}\right|$

**Proof.** Social welfare depends on the consumption of both low skilled and high skilled workers. The level of consumption in turn depends on the total wages. If *H* > *H*^∗^, then $\left(\frac{\partial {w}_t^h{h}_t}{\partial {h}_t}\right)>0$. This causes an increase in the consumption of high skilled agents. If $L>{L}^{\ast },\left(\frac{\partial {w}_t^l{l}_t}{\partial {l}_t}\right)<0$. This causes a decrease in the consumption of low skilled agents. If $\left|\frac{\partial {w}_t^l{l}_t}{\partial {l}_t}\right|>\left|\frac{\partial {w}_t^h{h}_t}{\partial {h}_t}\right|$ then the decrease in the consumption of low skilled agents is larger than the increase in the consumption of high skilled agents. This decreases social welfare.

## Intuition

This section elaborates on the intuition of the relationship between these health crises and income inequality. We expect that epidemics and pandemics cause an increase in income inequality. This is because these health crises cause authorities and policy makers to interfere in an attempt to contain an outbreak before it strains or overwhelms the health care system. Examples of these interventions are stay-at-home orders, shelter-in-place orders, restrictions imposed on in-person transactions, lockdowns and social distancing. These policies can cause the loss of jobs that cannot be done remotely but require in-person presence in the workplace. Many of these jobs are likely to be low-skilled jobs. On the other hand, many high-skilled jobs can be done remotely in an easier manner. Not to mention that high-skilled workers are more capable than low-skilled ones to use the technology that allows them to work remotely. Accordingly, more low-skilled jobs are lost during an epidemic or a pandemic than high-skilled ones. This can cause an increase in income inequality between low-skilled and high-skilled workers.

The health effects of epidemics and pandemics are also disproportionate between income categories. The fatality rate of epidemics or pandemics is usually highest amongst those who are less healthy due to obesity, worse diets or preexisting chronic conditions. These conditions are more common amongst the poor than the affluent. Thus, epidemics and pandemics have disproportionate health consequences that can exacerbate income disparities as well. This is because those who are affected can either die leaving their families without sufficient financial support, or survive but are unable to work for a period of time which adversely affects the family’s income. On the other hand, preventive care and public health education have steadily tilted towards the educated and the well-off. These groups tend to react to the epidemics in a manner that better enables them to lessen its spread compared to those who are poorer and less educated. These factors contribute to an increase in income inequality. Epidemics also tend to spread faster in densely populated areas which is typical of poorer communities, compared to the sparsely populated suburbs which only the affluent can afford. This implies that infectious diseases hit poorer areas harder than others. This causes the existing income inequality to exacerbate. The fatality rate of these health crises are also higher amongst the elderly who are more likely to have a larger accumulation of wealth and who may leave bequests to their children. This can increase wealth disparities especially if they have fewer offsprings on average.

Epidemics and pandemics also have an effect on the school system which is likely to affect worker’s compensation that is usually dependent on the level of educational attainment. School closures during an epidemic or a pandemic can widen the student achievement gap. Students from a poorer background are less likely to catch-up in an online educational or remote learning environment compared to their more affluent peers. This could be due to the lack of access to internet or the technological devices needed, the lack of knowledge of the technology used, or the need for both parents to work. This achievement gap in education causes a subsequent income gap. In addition, the policy response to epidemics and pandemics usually causes severe budget deficits that may lead to spending cuts on public education. This adversely affects the prospects of social upward mobility through education, and thus can cause persistence in income inequality. This implies that epidemics and pandemics can also have a long term effect on income inequality.

The economic conditions during epidemics and pandemics also favor larger corporations who can adjust their operations to serve their customers online and to deliver their commodities to the consumers’ doorsteps. These big businesses, compared to smaller ones, are also the ones who have sufficient cash buffer to ride out the economic repercussions caused by these health crises. On the other hand, closures of non-essential businesses during lockdowns can cause the shutdown of many small businesses that cannot serve their customers online. This may increase income inequality between shareholders of large corporations and small business owners.

The lower interest rates, due to monetary expansion in response to an epidemic-induced economic slowdown, can lower mortgage rates and increase mortgage applications. This increase in demand for houses can lead to a subsequent increase in house prices. The epidemics and pandemics can also increase the need to relocate to the suburbs away from densely populated urban areas that usually have higher rates of transmission. This also increases the demand for houses. The subsequent appreciation in the value of houses and real estate prices, which is one of the main sources of wealth, can increase wealth inequality between home owners and others. Lower interest rates can also lead to an increase in demand for stocks which could boost stock prices. This can also exacerbate wealth inequality between portfolio owners and others.

## Methods

This paper aims to empirically test our theoretical model on the effect of epidemics and pandemics on income inequality. The main study design is a cross-country econometric comparison using panel data. The setting of the study is global in nature as we use data for most countries in the world. In this section we describe the data for the dependent variable (income inequality) and the main variable of interest (epidemics dummy and deaths per 100,000 population), as well as control variables. Then we describe the econometrics methods including fixed effects and three-stage least squares regression analyses.

### Data

This paper uses a panel of 191 countries during the period 2000–2020. The countries included in the analysis are: Afghanistan, Albania, Algeria, Angola, Anguilla, Antigua and Barbuda, Argentina, Armenia, Australia, Austria, Azerbaijan, Bahamas, The Bahrain, Bangladesh, Barbados, Belarus, Belgium, Belize, Benin, Bhutan, Bolivia, Bosnia and Herzegovina, Botswana, Brazil, Brunei Darussalam, Bulgaria, Burkina Faso, Burundi, Cabo Verde, Cambodia, Cameroon, Canada, Central African Republic, Chad, Chile, China, Colombia, Comoros, Congo, Democratic, Republic of the Congo, Republic of Costa Rica, Croatia, Cyprus, Czech Republic, Cote d’Ivoire, Denmark, Djibouti, Dominica, Dominican Republic, Ecuador, Egypt, El Salvador, Equatorial Guinea, Estonia, Ethiopia, Fiji, Finland, France, Gabon, Gambia, The Georgia, Germany, Ghana, Greece, Grenada, Guatemala, Guinea, Guinea-Bissau, Guyana, Haiti, Honduras, Hong Kong SAR, Hungary, Iceland, India, Indonesia, Iran, Iraq, Ireland, Israel, Italy, Jamaica, Japan, Jordan, Kazakhstan, Kenya, Kiribati, Korea, Kosovo, Kuwait, Kyrgyz Republic, Lao P.D.R., Latvia, Lebanon, Lesotho, Liberia, Libya, Lithuania, Luxembourg, Macedonia FYR, Madagascar, Malawi, Malaysia, Maldives, Mali, Malta, Mauritania, Mauritius, Mexico, Micronesia, Moldova, Mongolia, Montenegro, Rep. of, Morocco, Mozambique, Myanmar, Namibia, Nauru, Nepal, Netherlands, New Zealand, Nicaragua, Niger, Nigeria, Norway, Oman, Pakistan, Palau, Panama, Papua New Guinea, Paraguay, Peru, Philippines, Poland, Portugal, Puerto Rico, Qatar, Romania, Russia, Rwanda, Samoa, Saudi Arabia, Senegal, Serbia, Seychelles, Sierra Leone, Singapore, Slovak Republic, Slovenia, Solomon Islands, Somalia, South Africa, South Sudan, Spain, Sri Lanka, St. Kitts and Nevis, St. Lucia, St. Vincent and the Grenadines, Sudan, Suriname, Swaziland, Sweden, Switzerland, Syria, Sao Tome and Principe, Taiwan, Tajikistan, Tanzania, Thailand, Timor-Leste, Togo, Tonga, Trinidad and Tobago, Tunisia, Turkey, Turkmenistan, Tuvalu, Uganda, Ukraine, United Arab Emirates, United Kingdom, United States, Uruguay, Uzbekistan, Vanuatu, Venezuela, Vietnam, West Bank and Gaza, Yemen, Zambia, and Zimbabwe. The summary statistics of the variables used in the analysis are included in Table 1.

**Table 1:**
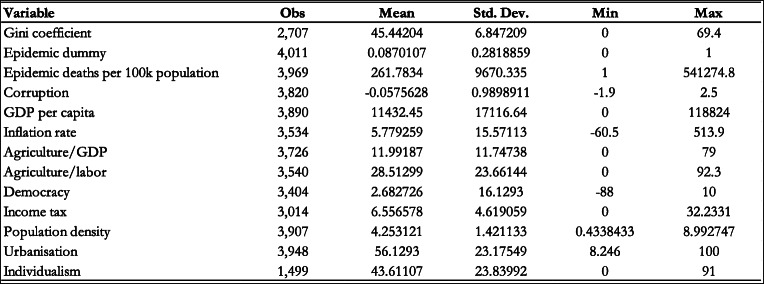
Summary Statistics

#### Epidemics and pandemics

Data on epidemics was compiled from the disease profiles in the World Health Organization.^1^ The World Health Organization Data and Statistics are used to identify the occurrence, location and time of the epidemic. This dataset includes, SARS (2002–04),^2^ H1N1 (2009),^3^ MERS-Cov (2012),^4^ Ebola (2013–2016)^5^ and SARS-Cov-2 (2020), the virus that causes COVID-19. Using this information, we construct a dummy variable as follows: if a country had a disease profile for that particular epidemic, a value of 1 was assigned; if it did not have a disease profile for the epidemic, a value of zero was assigned. Thus, the dummy variable takes the value of 1 for countries which had reported an epidemic during a year, and zero otherwise. For each of the epidemics we consider, only countries which had been affected by a given epidemic would have a disease profile. These are infectious disease epidemics that are highly contagious and have the potential to become pandemics. For COVID-19, all countries in the sample are affected. We also calculate the number of deaths per 100,000 population due to the epidemic or pandemic as another health indicator.^6^

#### Income inequality

The data for income inequality is derived from the Standardized World Income Inequality SWIID Database, Version 8.^7^ The SWIID’s income inequality estimates are based on reported Gini indices from published sources, including the OECD Income Distribution Database, the Socio-Economic Database for Latin America and the Caribbean generated by CEDLAS and the World Bank, Eurostat, the World Bank’s PovcalNet, the UN Economic Commission for Latin America and the Caribbean, national statistical offices around the world, and academic studies. The SWIID incorporates comparable Gini indices of disposable and market income inequality for 198 countries for as many years as possible from 1960 to the present.

#### Controls

Several control variables are used in the analysis to check the robustness of our results. These are factors that are identified by the literature as potential determinants of income inequality. The first variable used is Real Gross Domestic Product GDP per capita which is derived from the Penn World Tables version 8.0. The logarithm of real Gross Domestic Product per capita is used in the analysis. According to the Kuznets curve, there is an association between the level of economic development and the extent of income inequality.

We also use indicators that reflect the sectoral structure of the economy. In particular, we use the share of agriculture in the labor force and the share of agriculture in GDP. The first variable is defined as Agriculture, forestry, and fishing, value added (% of GDP). The second variable is defined as Agriculture, forestry, and fishing, value added per worker (constant 2010 US$). This data is derived from the World Development Indicators. We expect that an economy that is more agricultural in nature to have a lower level of income inequality.

We use various institutional quality indicators. Democracy reflects the quality of political institutions. The democracy variable is extracted from the Polity IV Project. The Polity score captures a country’s political regime on a 21-point scale ranging from − 10 (strongly autocratic) to + 10 (strongly democratic). The paper uses the Polity2 variable which is a modified version of the Polity variable by applying a simple treatment to convert instances of “standardized authority scores” (−66, −77, −88) to conventional polity scores within the range between − 10 to + 10. Some studies argue that a transition to democracy is expected to give greater weight to the preferences of the poor in collective decision-making. The poor may use the political process to their benefit and influence policy makers to implement inequality-reducing policies. Acemoglu et al. [1] survey the existing empirical literature on why democracy is expected to increase redistribution and decrease income inequality. Another institutional indicator is the corruption perception index derived from Transparency International. Some studies find an association between corruption and income inequality, such as Pedersen [21] and Spinesi [23].

We also include some policy indicators that reflect fiscal policy, monetary policy and trade policy. We include the tax rate in a country, derived from the World Development Indicators. The higher the tax rate the larger is the welfare state, which is expected to decrease income inequality. Another policy indicator is the inflation rate, consumer prices (%) derived from the World Development Indicators. This indicator reflects the efficiency of the conduct of monetary policy by central banks. Several studies examine the effect of inflation on income inequality. For instance, Albanesi [2] provide cross-country evidence on a positive association between inflation and income inequality. We also include trade openness, which is the sum of exports and imports of goods and services as a share of Gross Domestic Product. This is derived from the World Development Indicators. Some studies find an association between trade liberalization and income inequality, such as Goldberg and Pavcnik [15].

### Estimation

#### Results and robustness

This section empirically estimates the effect of epidemics and pandemics on income inequality for the period 2000–2020 as follows

12 $$ Gin{i}_{it}=\alpha +\beta EpiPa{n}_{it-1}+{X}_{it-1}\gamma +{\delta}_i+{\varepsilon}_t+{u}_{it} $$

where *Gini*_*it*_ is the Gini coefficient in country *i* in year *t*. *EpiPan*_*it* − 1_ is the indicator for epidemics or pandemics in country *i* in year *t* − 1. *X*_*it* − 1_ is a vector of control variables identified by the literature as determinants of income inequality in country *i* in year *t* − 1. The *δ*_*i*_ denotes a full set of country dummies, the *ε*_*t*_ denotes a full set of time effects that capture common shocks to income inequality of all countries, and *u*_*it*_ is an error term capturing all other omitted factors, with *E*(*u*_*it*_) = 0 for all *i* and *t*.

The fixed effects estimation results are included in Table 2 when our variable of interest is the epidemics dummy. Column 1 of Table 2 shows the results without control variables. We add the interaction term between the dummy variable and population density in the second column. This is to distinguish between the effect of epidemics and pandemics on income inequality in low and high levels of population density. The intuition is that the effect of these health crises is exacerbated with high population density. We also add the control variables in subsequent columns. The results in column 1 show that the dummy variable has a statistically significant and positive effect on income inequality. However, when we add the control variables, the dummy variable loses its statistical significance.

**Table 2:**
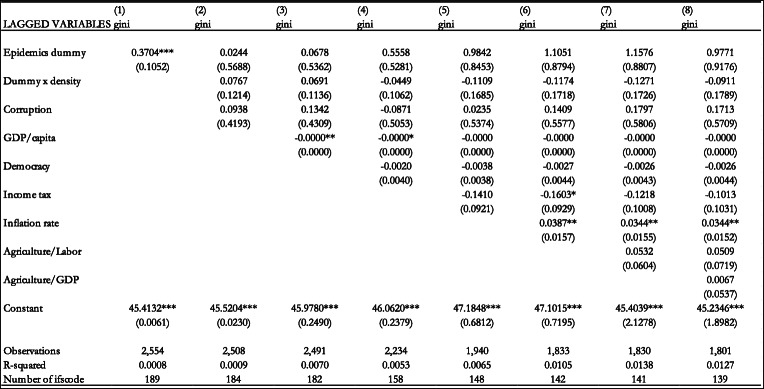
Effect of epidemics dummy on the Gini coefficient (Fixed Effects estimation). Notes: Standard errors in parentheses *** *p* < 0.01, ** *p* < 0.05, * *p* < 0.1

Table 3 includes the results when our variable of interest is the number of deaths per 100,000 population. The results in column 1 show that this variable has a weak statistically significant positive effect on income inequality. When we add the control variables in subsequent columns, the number of deaths per 100,000 population and the interaction term do not have a statistically significant effect on income inequality.

**Table 3:**
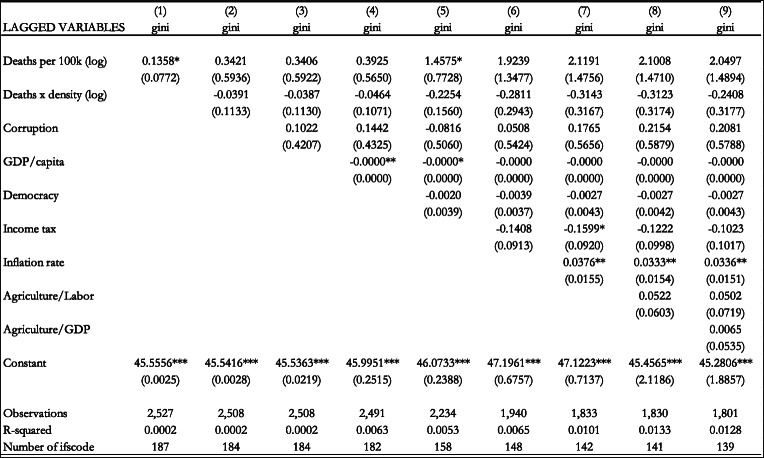
Effect of the epidemics deaths per 100,000 population (logs) on the Gini coefficient (Fixed Effects estimation). Notes: Standard errors in parentheses *** *p* < 0.01, ** *p* < 0.05, * *p* < 0.1

In Table 4, we include both the epidemics dummy and the number of deaths per 100,000 population. The estimation results show that the dummy variable has a statistically significant and positive coefficient in all specifications, while the number of deaths per 100,000 population is not statistically significant. This implies that it is the occurrence of the pandemic, as captured by the dummy variable, that triggers the policy response that may have an effect on income inequality, rather than its health consequences captured by the number of deaths per 100,000 population.

**Table 4:**
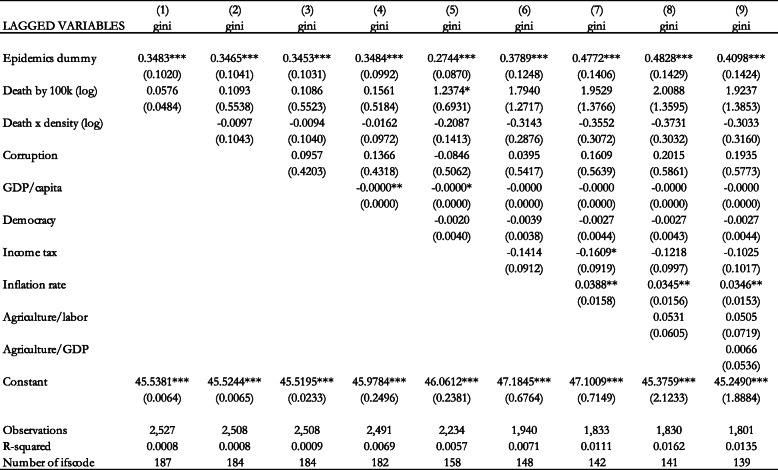
Effect of epidemics dummy and epidemics deaths per 100,000 population (logs) on the Gini coefficient (Fixed Effects estimation). Notes: Standard errors in parentheses *** *p* < 0.01, ** *p* < 0.05, * *p* < 0.1

In Table 5 we include both health variables with the exclusion of Ebola. This is because Ebola has different transmission mechanisms than the other epidemics considered in our paper. Other epidemics are diseases of the respiratory system, with similar transmission mechanisms that require similar policy responses. On the other hand, Ebola is transmitted through contact with bodily fluids. Thus, the reponse is different than those required for respiratory system illnesses. The results in Table 5 confirm our previous finding that the dummy variable has a statistically significant and positive coefficient, while the number of deaths per 100,000 population is not statistically significant in all specifications.

**Table 5:**
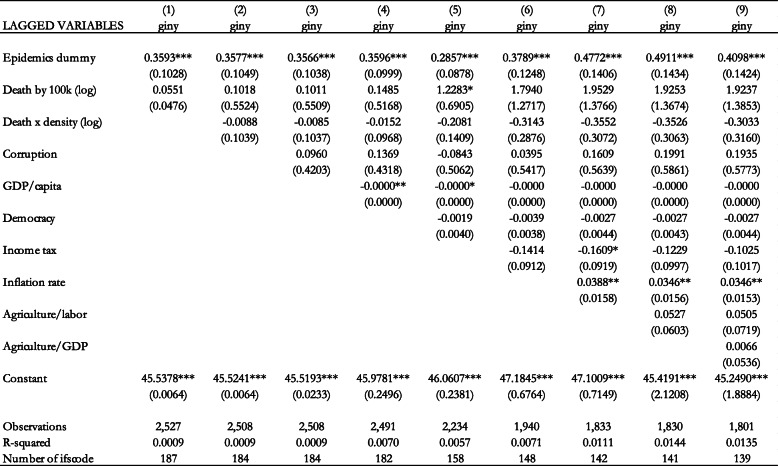
Effect of epidemics dummy and epidemics deaths per 100,000 population (logs) on the Gini coefficient (Fixed Effects estimation without Ebola). Notes: Standard errors in parentheses *** *p* < 0.01, ** *p* < 0.05, * *p* < 0.1

In order to incorporate the effect of the COVID-19 pandemic, we consider the contemporaneous effect of these health indicators on income inequality. This allows for the inclusion of the available data on COVID-19 in the year 2020. Table 6 shows that the dummy variable has a statistically significant and positive effect on income inequality in all specifications, while the death per 100,000 population indicator does not have a robust effect. Table 7 shows the estimation of the contemporaneous effect without Ebola. The results also confirm our finding of a statistically significant positive coefficient for the dummy variable, but not for the death per 100,000 population.

**Table 6:**
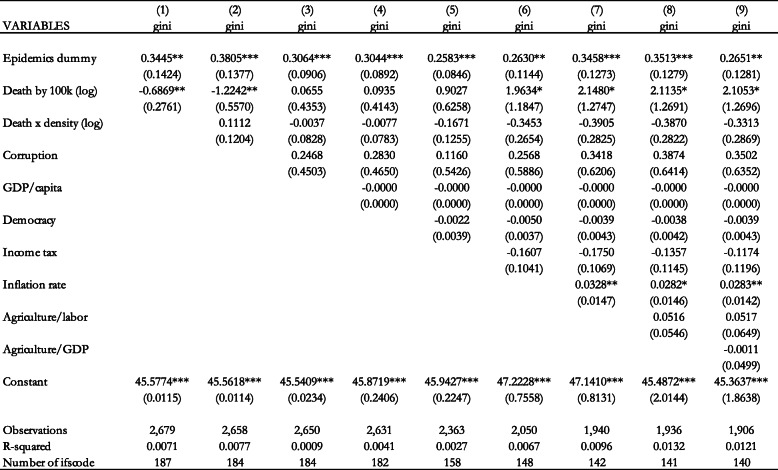
Contemporaneous effect of epidemics dummy and epidemics deaths per 100,000 population (logs) on the Gini coefficient (Fixed Effects estimation). Notes: Standard errors in parentheses *** *p* < 0.01, ** *p* < 0.05, * *p* < 0.1

**Table 7:**
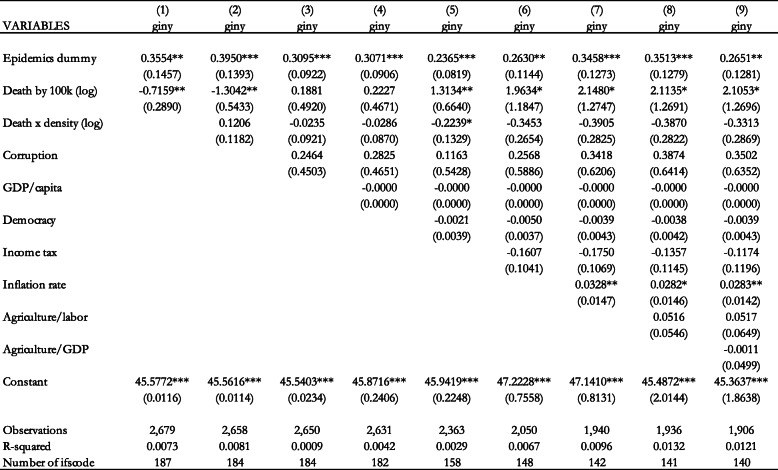
Contemporaneous effect of epidemics dummy and epidemics deaths per 100,000 population (logs) on the Gini coefficient (Fixed Effects estimation without Ebola). Notes: Standard errors in parentheses *** *p* < 0.01, ** *p* < 0.05, * *p* < 0.1

#### Causality

This section attempts to address the issue of causality between these health crises and income inequality. As much as epidemics and pandemics can affect income distribution as stated in our theoretical setup and the intuition sections, the extent of income inequality can determine the disproportionate exposure of various groups to a health crisis causing it to exacerbate to an epidemic or a pandemic. At high levels of income inequality, there is a large group of low income individuals that experience a higher level of exposure, transmission and mortality in a health crisis. This could be due to the fact that this group typically have worse pre-existing health conditions, have less health insurance coverage, and have less education and awareness of health issues. This can cause income inequality to lead to an exacerbation of a health crisis causing it to turn into an epidemic or a pandemic. An article in the New York Times supports this view and suggests that^8^: “*those in lower economic strata are likelier to catch the disease...... At the same time, inequality itself may be acting as a multiplier on the coronavirus’s spread and deadliness.*”

This implies an issue of reverse causality. To deal with potential endogeneity, we use a system of simultaneous equations that can be jointly estimated using Three-Stage-Least-Squares (3SLS). Simultaneous equations are a statistical model in which the dependent variables are functions of other dependent variables, rather than just independent variables. In our context, both the Gini coefficient and the epidemic and pandemic indicator can be determined jointly as follows

$$ Gin{i}_{it}=\alpha +\beta EpiPa{n}_{it-1}+{X}_{it-1}\gamma +{u}_{it} $$

13 $$ EpiPa{n}_{it}=\lambda +\tau Gin{i}_{it-1}+{Z}_{it-1}\rho +{e}_{it} $$

where *Z*_*it* − 1_ is a vector of control variables identified by the literature as determinants of an epidemic or a pandemic in country *i* in year *t* − 1. This vector includes population density, urbanization, democracy and individualism. The identification strategy is based on the intuition that a high level of population density in a country can contribute to a faster spread of a disease causing it to turn into an epidemic, and can also exacerbate the health effects of these epidemics. Population density is captured by the number of people per square kilometer of land area, derived from the World Development Indicators. Infectious diseases also spread faster in urban concentrations compared to sparsely-populated rural areas. Thus, the urbanization rate is expected to have a positive association with the epidemics and pandemics indicators. Urbanization is measured by the urban population as a percentage of total population, derived from the World Development Indicators. We also include our indicator for democracy, the Polity score. Democratic governments are more likely to be held accountable for how they deal with health crises and accordingly can be more proactive in their attempt to contain the spread of infectious diseases. On the other hand, authoritarian countries tend to cover up the spread of a disease, which makes them more impotent to deal with an epidemic once out of control. Finally, we include the individualism score from the Geert Hofstede dataset.^9^ This is because those in collectivist cultures are more likely to sacrifice their personal freedom to follow the rules imposed for the common good of the group. While those in individualistic cultures value more their individual freedoms and personal rights even in the face of a calamity that requires the collective involvement of every individual to be able to deal with it. Thus, we expect that individualism to have a positive association with the spread of infectious diseases which can transform them into epidemics or pandemics. In our estimation, we include an interaction term between population density and individualism.

In this context, we do not consider the effect of the epidemic dummy since this can complicate the estimation when a dummy is the dependent variable in the second equation. The results of the Three-Stage-Least-Squares estimation are included in Table 8 when our variable of interest is the number of deaths per 100,000 population. In the table, column 1 shows the results of the first equation where the Gini coefficient is our dependent variable while column 2 shows the results of the second equation where the health indicator is the dependent variable. The results show that the health indicator does not have a statistically significant effect on the Gini coefficient, while income inequality has a statistically significant negative effect on the health indicator in the 2020 sample. The results also show that the interaction term is not statistically significant either.

**Table 8:**
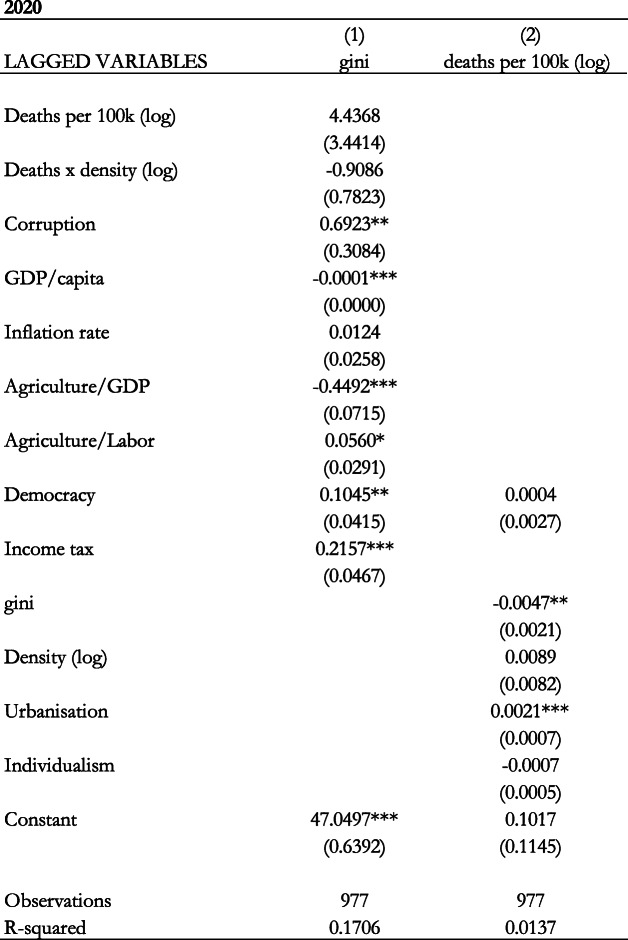
Effect of epidemic deaths per 100,000 population (logs) on the Gini Coefficient (Three-Stage-Least-Squares). Notes: Standard errors in parentheses *** *p* < 0.01, ** *p* < 0.05, * *p* < 0.1

We also conduct the Three-Stage-Least-Squares estimation to examine the contemporaneous effect in Table 9, with and without Ebola. This is to examine the effect of COVID-19 given the data limitations. Columns 1 and 2 include the results with Ebola, while columns 3 and 4 include the results without Ebola. In each case, the first column shows the results of the first equation where the Gini coefficient is our dependent variable while the second column shows the results of the second equation where the health indicator is the dependent variable. The results show that the deaths per 100,000 population has a positive effect on the Gini coefficient. This effect is statistically significant and economically important as can be seen from the magnitude of the coefficient.

**Table 9:**
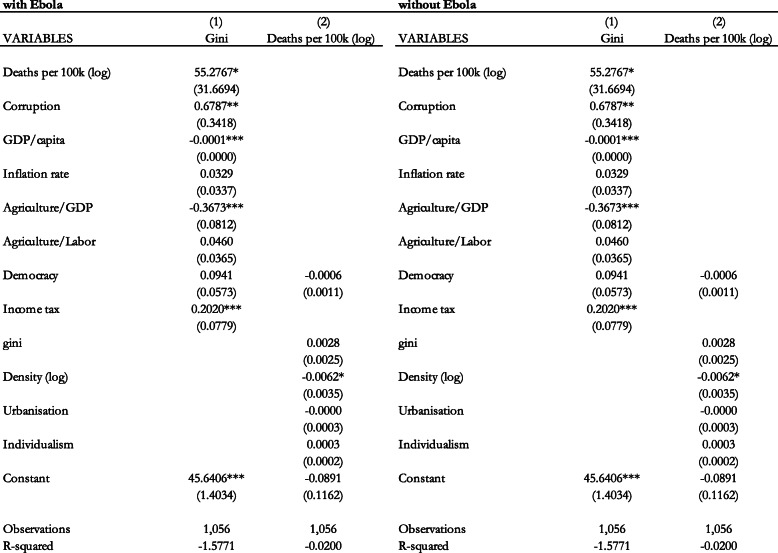
Contemporaneous effect of epidemic deaths per 100,000 population (logs) on the Gini coefficient (Three-Stage-Least-Squares with and without Ebola). Notes: Standard errors in parentheses *** *p* < 0.01, ** *p* < 0.05, * *p* < 0.1

This implies that before COVID-19 it was the policy response to these health crises that mattered in its effect on income inequality, When we include the effect of COVID-19, and addressing potential endogeneity, the health consequences of these epidemics and pandemics also matter for income inequality. This suggests that not only the occurrence, captured by the dummy variable, but also the health consequences of COVID-19, captured by the number of deaths, have a significant effect on income inequality.

## Discussion

Our findings are consistent with other recent research. For example, years spent in poverty were estimated for the pandemic: it had generated at least 68 million additional poverty years in 150 countries [8]. That result is consistent with within-country inequality, even if it may not be consistent with cross-country global income inequality [7]. Similarly, research from Italy concludes that the positive shift in working from home (WFH) feasibility is associated with an increase in average labor income, yet the benefit is not equally distributed among employees: the increased opportunity of WFH favors male, older, high-educated, and high-paid employees [4].

Our 3SLS approach allows for causal interpretation from epidemics and pandemics to income inequality. This is important because the reverse association has also been documented for OECD countries: A 1% increase in the Gini coefficient is associated with a 4% increase in COVID-19 cases per-million and a 5% increase in deaths per-million [25]. Similar results were found for French departments, where the authors conclude “inequality kills: departments with higher income inequality experience more deaths, more discharged (gravely ill) patients and more infections” [13]. And similarly for U.S. counties, where income inequality increases the infection rate, yet the effects are largely accounted by the correlation with racial composition [6].

Future research should consider the structural factors leading to the observed results. This is particularly pertinent to areas of high income inequality, such as Latin America, where recent research found that most of the dispersion in labor income loss across countries is explained by sectoral/occupational employment structure of the economies [9]. Future research can also include additional data on income inequality to examine the effect of lagged health indicators including COVID-19 on income inequality.

## Conclusion

This paper tests a theoretical model that predicts that epidemics and pandemics increase income inequality. To achieve its objective, the paper explores the effect on the Gini coefficient of a dummy variable that indicates the occurrence of an epidemic or a pandemic in a country in a given year and the number of deaths per 100,000 population. The fixed effects estimation shows a robust statistically significant positive effect of the dummy variable but an insignificant effect of the number of deaths per 100,000 population. The interpretation is that it is the occurrence of a pandemic, captured by the dummy variable, that is what triggers the policy response that may affect income inequality, rather than the health consequences of these crises captured by the number of deaths per 100,000.

To properly address potential endogeneity, we implement a Three-Stage-Least Squares estimation technique. The 3SLS estimation shows that the number of deaths per 100,000 population variable has a statistically significant contemporaneous positive effect on the Gini coefficient, especially when we incorporate the data on COVID-19. This suggests that not only the occurrence but also the health consequences of COVID-19 have a significant effect on income inequality.

## Data Availability

The datasets analysed during the current study is available from the corresponding author on reasonable request.

## Abbreviations

WFH: Working from home feasibility
SWIID: Standardized World Income Inequality
3SLS: Three-Stage-Least-Squares
GDP: Real Gross Domestic Product
SARS: Severe acute respiratory syndrome
MERS: Middle East Respiratory Syndrome

## Footnotes

1 https://www.who.int/data/collections

2 https://www.who.int/publications/m/item/summary-of-probable-sars-cases-with-onset-of-illness-from-1-november-2002-to-31-july-2003

3 https://www.who.int/csr/disease/swineflu/updates/en/

4 http://www.emro.who.int/pandemic-epidemic-diseases/mers-cov/mers-situation-update-january-2020.html

5 https://apps.who.int/ebola/current-situation/ebola-situation-report-16-march-2016

6 https://coronavirus.jhu.edu/data/mortality

7 https://dataverse.harvard.edu/dataset.xhtml?persistentId=doi:10.7910/DVN/LM4OWF

8 https://www.nytimes.com/2020/03/15/world/europe/coronavirus-inequality.html

9 https://geerthofstede.com/research-and-vsm/dimension-data-matrix/
